# DARKIN: a zero-shot benchmark for phosphosite–dark kinase association using protein language models

**DOI:** 10.1093/bioinformatics/btaf480

**Published:** 2025-10-29

**Authors:** Emine Ayşe Sunar, Zeynep Işık, Mert Pekey, Ramazan Gökberk Cinbiş, Oznur Tastan

**Affiliations:** Faculty of Engineering and Natural Sciences, Sabanci University, Istanbul 34956, Türkiye; Faculty of Engineering and Natural Sciences, Sabanci University, Istanbul 34956, Türkiye; Faculty of Engineering and Natural Sciences, Sabanci University, Istanbul 34956, Türkiye; Department of Computer Engineering, Middle East Technical University, Ankara 06800, Türkiye; Faculty of Engineering and Natural Sciences, Sabanci University, Istanbul 34956, Türkiye

## Abstract

**Motivation:**

Protein language models (pLMs) have emerged as powerful tools for capturing the intricate information encoded in protein sequences, facilitating various downstream protein prediction tasks. With numerous pLMs available, there is a critical need for diverse benchmarks to systematically evaluate their performance across biologically relevant tasks. Here, we introduce DARKIN, a zero-shot classification benchmark designed to assign phosphosites to understudied kinases, termed dark kinases. Kinases, which catalyze phosphorylation, are central to cellular signaling pathways. While phosphoproteomics enables the large-scale identification of phosphosites, determining the cognate kinase responsible for the phosphorylation event remains an experimental challenge.

**Results:**

In DARKIN, we prepared training, validation, and test folds that respect the zero-shot nature of this classification problem, incorporating stratification based on kinase groups and sequence similarity. We evaluated multiple pLMs using two zero-shot classifiers: a novel, training-free k-NN-based method, and a bilinear classifier. Our findings indicate that ESM, ProtT5-XL, and SaProt exhibit superior performance on this task. DARKIN provides a challenging benchmark for assessing pLM efficacy and fosters deeper exploration of under-characterized (dark) kinases by offering a biologically relevant test bed.

**Availability and implementation:**

The DARKIN benchmark data and the scripts for generating additional splits are publicly available at: https://github.com/tastanlab/darkin

## 1 Introduction

Building on the success of large language models in natural language processing (Yupeng *et al.* 2024), protein language models (pLMs) have been developed to capture the complex information embedded within protein sequences (Rao *et al.* 2019, Elnaggar *et al.* 2021, Lin *et al.* 2023, Meier *et al.* 2021, Lin *et al.* 2022, Brandes *et al.* 2022, Ferruz *et al.* 2022, Geffen *et al.* 2022, Elnaggar *et al.* 2023, Su *et al.* 2024, ESM Team, 2024, Fournier *et al.* 2024, Zhang *et al.* 2025, Yupeng Wang *et al.* 2024, Ouyang-Zhang *et al.* 2024, Hayes *et al.* 2025, Peng *et al.* 2025). By generating semantic representations of proteins, pLMs enable a broad range of sequence-based prediction tasks. However, as more pLMs become available, systematically benchmarking their performance is essential to determine their reliability and applicability in diverse biological contexts. Previous work has compared the pLMs in their ability to predict proteins’ functional properties (Unsal *et al.* 2022, Schmirler *et al.* 2024, Zhang *et al.* 2025) and functional motifs (Savojardo *et al.* 2023). In this work, we provide a novel biologically relevant zero-shot prediction benchmark for phosphosite–dark kinase associations and compare the pLMs in terms of their ability to capture intrinsic sequence properties within this challenging task.

Phosphorylation events are key regulators of protein function in signal transduction pathways, and their dysfunction is associated with many diseases (Gaestel *et al.* 2009, Müller *et al.* 2015, Wu *et al.* 2023). Kinases are the enzymes that catalyze the phosphorylation of other proteins in a target-specific manner (Hunter 1995). For this reason, kinases are major drug targets in diseases such as cancer, infectious diseases, and neurological disorders (Blume-Jensen and Hunter 2001, Cohen *et al.* 2021). Phosphorylation involves transferring a phosphate from adenosine 5’-triphosphate (ATP) to amino acid residues (Cohen 2002). These phosphorylated residues, referred to as *phosphosites*, are integral to modulating the protein’s structure and function.

**Figure 1.**
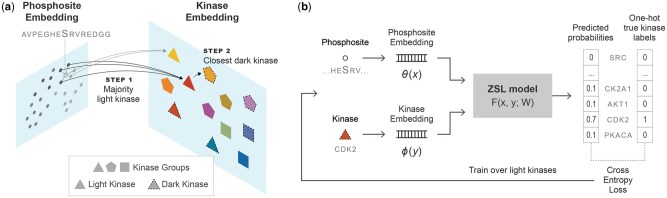
(a) k-NN-based zero-shot classifier. First, the test phosphosite’s nearest neighbor phosphosites are determined in the training data. The majority vote is taken among the neighbors’ class labels to pick the most likely light kinase. Then, the dark kinase most similar to this light kinase is picked. (b) The bilinear compatibility function *F* takes the phosphosite and kinase embedding vectors and is trained to minimize the cross-entropy loss over light kinases. At the prediction time, *F* is used to assess the compatibility of the phosphosite and the dark kinases.

Although high-throughput phosphoproteomics enables the identification of phosphosites at the proteome level, experimentally determining the kinase responsible for a phosphorylation event remains a major challenge. Notably, more than 95% of reported human phosphosites have no known cognate kinase (Needham *et al.* 2019), and 25% of the kinases are yet to be assigned to a phosphorylation event; for about 35% of the kinases, there are 1–10 phosphosites have been identified (Fig. 1, available as supplementary data at *Bioinformatics* online). Consequently, most of the phosphoproteome and the kinome are in the dark (Needham *et al.* 2019, Deznabi *et al.* 2020, Vella *et al.* 2022). Associating “orphan” phosphosites to their respective kinases is an important task that would help understand the biological function of these phosphorylation events and discover new drug targets (Needham *et al.* 2019, Deznabi *et al.* 2020, Berginski *et al.* 2021). In this work, given a phosphosite, we aim to predict the dark kinase associated with this phosphosite.

The contributions of this work can be summarized as follows: (i) We present a reproducible benchmark dataset for predicting dark kinase–phosphosite associations. The task is formulated as given a phosphosite, predict the associated dark kinase of that site. (ii)We propose a strategy to split the dataset into train, validation, and test splits for this zero-shot multi-class prediction task, with stratification based on kinase groups, the number of phosphosites per kinase, and kinase sequence similarity. (iii) We present a novel, training-free, k-NN-based zero-shot classification method for assessing the performance of pLMs under the task of predicting the dark kinase of a given phosphosite. (iv) We evaluate and compare various pLMs using two distinct zero-shot classification approaches.

## 2 Materials and methods

### 2.1 Problem description

Let X denote the space of phosphosite sequences and Y denote the set of all human kinases. The task of kinase–phosphosite association prediction involves identifying the kinase y∈Y most likely to catalyze the phosphorylation of a given phosphosite sequence x∈X. Since a phosphosite can be phosphorylated by multiple kinases, we frame the problem as a multilabel classification task. We denote the training kinases as $Y_{tr}\subset Y$ and the test kinases as $Y_{te}\subset Y$. The set $Y_{te}$ comprises the zero-shot classes, and the training and test kinase sets are disjoint. The training data, $D_{tr}=(x_{i},y_{i}),i=1,\ldots ,N_{tr}$, consists of pairings of train kinases with their associated phosphosites, where $y_{i}\in Y_{tr}$. Similarly, the test data contains phosphosite pairings of the test kinases $Y_{te}$.

### 2.2 Dataset curation and processing

The DARKIN dataset is built on human kinases and their associated phosphosites. Several publicly available human kinase lists are available, yet they partially overlap due to ambiguities in defining kinase domains. The most widely used and oldest list is the 518 human kinase set defined by Manning *et al.* (2002). Other sources, such as kinasecom (http://kinase.com/), Eid *et al.* (2017), The UniProt Consortium (2023), and Moret *et al.* (2020), provide alternative kinase lists with some variations. For the current work, we resort to an up-to-date list from Moret *et al.* (2020), which includes 557 human kinases, each containing at least one kinase domain.

We obtained experimentally validated kinase–phosphosite associations from the PhosphoSitePlus (Hornbeck *et al.* 2012) (downloaded in May 2023). Kinase–phosphosite associations, which are related to non-human kinases, are removed. We did not apply the same restriction to substrates, as substrates from the model organisms are used to probe the interactions. We removed kinase isoforms and fusion kinases and used the canonical form specified in the UniProt human proteome (Bairoch *et al.* 2005) (downloaded May 2023). Phosphosites are represented as 15-residue amino acid sequences, including seven residues flanking the phosphosite on both sides. Previous work has shown that phosphosite sequences of length 15 or shorter led to better performances (Trost and Kusalik 2011, Hornbeck *et al.* 2014, Wagih *et al.* 2015, Deznabi *et al.* 2020). Padding was applied to ensure the phosphosite remains centered when it is near the N or C terminus of the protein.

Protein sequences were retrieved from UniProt via the API (Ahmad *et al.* 2025) (accessed December 2023). If the substrate could not be uniquely mapped to a Uniprot ID, we removed all phosphosite–kinase associations of these substrates. We retrieved the kinase domain sequences using the domain indices provided in Moret *et al.* (2020). Kinases are categorized into groups and families by Manning *et al.* (2002) according to their domain sequence similarities. We retrieved the kinase family and group information from Moret *et al.* (2020). Missing group and family information was imputed according to their similarity to other kinases. We defined a kinase group Other2 and a kinase family otherFamily for kinases that cannot be assigned to a family or group due to their dissimilarity to the rest of the groups. Another categorical information regarding kinases is the Enzyme Commission (EC) categorization. EC numbers categorize kinases according to their functionality. We downloaded EC numbers of the kinases (downloaded July 2023) (Bairoch 2000). We obtained protein structure data from the AlphaFold Protein Structure Database using AlphaFoldAPI at EBI (https://alphafold.ebi.ac.uk) (Jumper *et al.* 2021, Varadi *et al.* 2022) and PDBe (Varadi *et al.* 2020). For isoform proteins lacking structural data in AlphaFold and PDBe, we used ColabFold to predict 3D structures (Mirdita *et al.* 2022).

### 2.3 Evaluated protein language models and baseline encodings

We selected pLMs whose models were accessible, reported to perform well in the literature, and were recent. Table 1 presents the pLMs we evaluated, along with their key properties. For more efficient processing, we computed the column-wise average of the embedding for all pLMs, excluding the vectors corresponding to the padding (PAD) token. For pLMs with a classification (CLS) token, we used the embeddings corresponding to this token to summarize the overall representation.

**Table 1.** The protein language models (pLMs) compared in this study.

PLM	Dataset	Vector size	Model size	Representation	Objective	Citation
TAPE	PFAM	768	38M	Sequence	Sequence-based, structural feature prediction	Rao *et al.* (2019)
ProtBERT	BFD100, UniRef100	1024	420M	Sequence	Sequence-based, structural, physicochemical feature prediction	Elnaggar *et al.* (2021)
ProtALBERT	UniRef100	4096	224M			
ProtT5-XL	BFD100	1024	3B			
ESM1B	UniRef50	1280	650M	Sequence	Structural, physicochemical feature prediction	Lin *et al.* (2023)
ESM1v	UniRef90	1280	650M	Sequence	Sequence variant prediction	Meier *et al.* (2021)
ESM2	UniRef50	1280	650M	Sequence	Structural feature, contact prediction	Lin *et al.* (2022)
ProteinBERT	UniRef90	1562	16M	Sequence	Sequence-based feature, GO annotation prediction	Brandes *et al.* (2022)
ProtGPT2	UniRef50	1280	738M	Subword	Protein design and engineering	Ferruz *et al.* (2022)
DistilProtBERT	UniRef50	1024	230M	Sequence	Sequence-based, structural, physicochemical feature prediction	Geffen *et al.* (2022)
Ankh	UniRef50	1536	1.5B	Sequence	General purpose modeling	Elnaggar *et al.* (2023)
SaProt	AlphaFold2, PDB	1280	650M	Sequence, structure	Structure-aware feature, mutation effect prediction	Su *et al.* (2024)
ESM3	UniRef, MGnify90, JGI, OAS PDB, InterPro, InterProScan	1536	1.4B	Sequence, structure, function	Protein generation	Hayes *et al.* (2025)
ESMC	UniRef, MGnify,JGI	1152	600M	Sequence	Sequence-based feature, contact prediction	ESM Team (2024)
ISM2	Uniclust30, PDB	1280	650M	Sequence	Sequence-based, structural, functional feature prediction	Ouyang-Zhang *et al.* (2024)
DPLM	UniRef50	960	650M	Sequence	Conditional and unconditional	Wang *et al.* (2024)
					Protein generation	
AMPLIFY	UniRef, OAS, SCOP, UniProt	1280	350M	Sequence	Structural feature, contact prediction	Fournier *et al.* (2024)
PTM-Mamba	UniProt Swiss-Prot PTM	768	220M (Mamba) + 650M (ESM2)^a^	Sequence	PTM-related prediction, PTM discovery	Peng *et al.* (2025)

a This is the parameter size of ESM2, which is also used in PTM-Mamba. The versions of the models are specified in Table 4, available as supplementary data at *Bioinformatics* online.

In addition to the pLM, we used the following encodings as the baseline representations:

- **One-hot encoding**: The input sequence is expressed as a binary vector of amino acids.
- **BLOSUM62 encoding**: The encoding uses the row corresponding to a particular amino acid in the BLOSUM62 matrix, which represents the probability of substitution of that amino acid by any other amino acid.
- **NLF encoding**: NLF captures the physicochemical properties of amino acids and is determined by a non-linear Fisher transform (Nanni and Lumini 2011). The representations are computed using the epitope prediction tool (Farrell 2021).
- **ProtVec**: ProtVec is a skip-gram neural network model trained to provide a continuous representation of protein sequences (Asgari and Mofrad 2015). ProtVec provides a 100-dimensional embedding for each 3 g, and the average embedding is used to represent the sequence.

### 2.4 Evaluation splits

In the zero-shot learning (ZSL) evaluation protocol, it is crucial to ensure class separation during model training and hyperparameter tuning (Xian *et al.* 2017). Therefore, examples are divided into train, validation, and test sets based on their associated class labels. In our earlier work, DeepKinZero evaluation Deznabi *et al.* (2020), we partitioned the data into training, validation, and test sets according to the number of phosphosites associated with each kinase. Kinases with more than five phosphosites were assigned as training classes, while kinases associated with exactly five phosphorylation sites were designated as validation kinases. The remaining kinases, each with fewer than five phosphosites, form the test or zero-shot classes. Thus, in this setup, the zero-shot kinases represent the dark kinases, whereas the training classes are light kinases. While this splitting strategy closely mirrors the real-world scenario of the deployed model, the limited number of examples for each class in the test set complicates the reliable estimation of evaluation performance. Therefore, we establish a setup where a portion of the well-studied kinases (light kinases) is held out as zero-shot classes and is excluded from the training process. Thus, imitating that light kinases are dark kinases. We follow this strategy to ensure that we have enough data from each kinase in the test set to report a more robust evaluation of the performance metrics. When creating the splits, we consider the following criteria to ensure a fair evaluation of data splits:

- **Number of phosphosites per kinase**: To ensure robust evaluation, we set a minimum threshold for the number of kinase–phosphosite pairs associated with each kinase in the test and validation sets. This prevents relying on very few data points related to a specific kinase class, minimizing inaccurate and unstable results. Thus, we invert the roles of light and dark kinases in evaluation: the test data include well-studied kinases (light kinases), while the training primarily comprises understudied kinases (dark kinases). However, it is crucial to note that this arrangement is solely for evaluation purposes; the deployed model can predict dark kinases.
- **Stratification based on kinase groups**: Kinases within the same kinase group share evolutionary relationships and functional similarities (Manning *et al.*, 2002). After preprocessing, the dataset contains only 392 kinases distributed across 11 kinase groups and 129 kinase families. Stratifying by kinase families is impractical due to the limited number of kinases per family, which would hinder equal representation of each kinase group in each split. Thus, we stratify kinases based on their group membership, ensuring the representation of kinase groups in the training, validation, and test sets whenever possible.
- **Sequence similarity of kinases**: In light of the inference task, which is to predict the kinase for a given phosphosite, to avoid optimistic performance estimates, kinases with sequence similarity are grouped and assigned exclusively to the same set (train, validation, or test). This criterion is important to prevent the model from being trained on kinases that are highly similar to the kinases in the test set, thereby avoiding optimistic evaluation results. It also aligns with the principles of ZSL by guaranteeing that all kinases in the test set are entirely new to the model. Sequence similarity is determined by sequence identity calculated after pairwise global alignment of the kinase domains.

Note that a single phosphosite can be targeted by multiple kinases, which may result in the same phosphosite appearing in both the training and test sets with different kinase labels. We quantified the multilabel nature of the task in Fig. 3, available as supplementary data at *Bioinformatics* online, which shows the number of sites associated with a single kinase or multiple kinases in each split. Additionally, we report the sites unique to the test set or shared with the validation and training data in the Fig. 4, available as supplementary data at *Bioinformatics* online. While a site-based split of the train, validation, and test set is possible, it is difficult to obtain a balanced split based on all four criteria. More importantly, it is not necessary, as the aim is to predict the right kinases for a known phosphosite. Even when a phosphosite appears in both splits, the associated kinase labels are disjoint across training and test sets. The model is still required to generalize to unseen kinases. It is indeed more challenging for the model, as it has previously associated this site with a different kinase and now needs to predict its association with the unseen test kinase. Therefore, this strategy does not affect the integrity of the evaluation process.

Taking all these aspects into consideration, we divided the dataset into training (80%), validation (10%), and test (10%) sets. We first categorize kinases as train or test kinases according to the number of phosphosites they are associated with. Kinases that are associated with fewer than 15 phosphosites are defined as train kinases. Later, kinases with at least 90% sequence identity are grouped and are randomly defined as entirely train or test kinases altogether. From the remaining kinases, test kinases are randomly selected from each kinase group in a stratified manner to ensure sufficient test example pairs from each kinase group. All remaining kinases are designated as train kinases. This process is repeated to determine validation kinases from among train kinases by setting the threshold for kinases in validation to be at least 10 phosphosites per kinase. Finally, the train, validation, and test sets include all train phosphosite–kinase pairs associated with the kinases in that relative set. Splitting the kinases into train, validation, and test is performed in a randomized and reproducible manner. Thus, different splits of the DARKIN dataset can be generated by setting different random seeds.

We evaluate our methods using the macro average precision (AP) score. AP summarizes the precision-recall curve at all recall levels (Salton and McGill 1983). In this way, AP provides a measure of how well the model is able to rank positive samples over negative samples. By using AP, for each kinase, we rank the prediction probabilities made for all phosphosite samples. If the model is able to assign relatively higher probabilities to phosphosites that are actually known to be phosphorylated by that kinase (the ground-truth phosphosites for that kinase), then we obtain higher scores closer to 1, indicating that the model ranks positive sites above negative ones, and hence achieves higher AP scores. In our setup, we calculate the AP score for each kinase and then take the mean across all kinase classes, hence calculating the macro AP. Although top-k accuracy is a well-known metric, in our setting it fluctuated sharply—small changes in predictions for the sparsely represented kinase classes produced large jumps in the score. To counter this instability and the effects of class imbalance, we report *macro* AP, which assigns equal weight to each kinase class. Macro AP, therefore, provides a steadier assessment of performance across both common and rare classes. When multiple kinases can phosphorylate a phosphosite, we accept the predicted kinase as a true positive if it matches any of the true kinases associated with it.

### 2.5 Zero-shot classifiers

We employ two ZSL models in our experiments. The first is a fitting-free method based on an adapted k-NN classifier, intentionally kept simple. The second model is a well-established bilinear zero-shot compatibility model. Further details on these approaches are provided in the following sections.

#### 2.5.1 Zero-shot k-NN classifier

To benchmark the zero-shot dark kinase prediction performance, we devised a simple baseline method by adapting the principles of the k-NN algorithm for supervised classification to our zero-shot classification task. For a given test phosphosite, we first locate the *k* most similar training phosphosites in the phosphosite representation space. Subsequently, we identify the most common *light kinase* among the kinases associated with the nearest neighbor phosphosites. In cases where there is no majority, we choose the nearest neighbor’s light kinase. Unlike the supervised k-NN approach, we predict the dark kinase that most resembles the predicted light kinase in the representation space. Kinase similarity is assessed using the cosine similarity of the kinase embedding vectors. These cosine similarity scores are considered our prediction scores, indicating how likely each dark kinase is to phosphorylate the test phosphosite at hand. This procedure is depicted in Fig. 1a.

Our motivation for devising this method is to evaluate the pLMs as *directly* as possible, in the sense that the approach does not involve numerical optimization, and the only hyperparameter is *k*. This simplicity provides an additional view of the relative strengths of the pLMs, largely avoiding model selection effects.

#### 2.5.2 Bilinear zero-shot learning model

The second ZSL method we use is a bilinear compatibility model. While a variety of other ZSL methods, particularly in image classification, have been proposed over the years, variants based on bilinear compatibility models are arguably among the most established (Frome *et al.* 2013, Romera-Paredes and Torr 2015, Akata *et al.* 2015, Akata *et al.* 2016, Xian *et al.* 2017, Kodirov *et al.* 2017, Sumbul *et al.* 2018, Deznabi *et al.* 2020). Therefore, they are particularly suitable for our pLM evaluation purposes.

The bilinear zero-shot model (BZSM) aims to estimate the compatibility between a given pair of phosphosite *x* and kinase *y* (illustrated in Fig. 1b). In our work, we use the formulation variant proposed and used in (Sumbul *et al.* 2018, Deznabi *et al.* 2020), which defines the compatibility function $F(x,y)=[\theta {\left(x\right)}^{⊤}\;1]W{\left[\phi {\left(y\right)}^{⊤}\;1\right]}^{⊤}$ where $\theta (x)\in R^{d}$ is the phosphosite representation, and $\phi (y)\in R^{m}$ is the kinase representation. The augmentation of both representations with separate bias dimensions increases the expressivity of the model (Sumbul *et al.* 2018), which can more clearly be observed when the definition is expanded:


(1) $$F(x,y)=\theta {\left(x\right)}^{⊤}W\phi (y)+\theta {\left(x\right)}^{⊤}W_{\cdot ,m}+W_{d,\cdot }\phi (y)+W_{d+1,m+1}.$$

In this formulation, the first term estimates pairwise compatibility. The second term acts analogously to a log p(x) prior, formulated via a linear estimator conditioned on θ(x). Similarly, the third term is a log p(y) prior, expressed as a linear function of ϕ(y). And finally, the last term is simply a trainable scalar. The model is trained by minimizing the regularized cross-entropy loss:


(2) $$\underset{W}{\min}-\sum_{(x,y)\in D_{tr}}\;\text{log}\;p(y|x)+\lambda ||W|{|}^{2}$$

where the summation runs over all phosphosite–kinase pairs available in the training set $D_{tr}=(x_{i},y_{i})$, and p(y|x) is the softmax of *F* over the light kinases:


(3) $$p(y|x)=\frac{\;\text{exp}\;F(x,y)}{\sum_{y^{\prime }\in Y_{tr}}\;\text{exp}\;F(x,y^{\prime })}.$$

The $\ell_{2}$ regularization term in Equation (2) is implemented as *weight decay* in practice. At test time, p(y|x) is calculated via softmax over the test kinases.

## 3 Results

### 3.1 Hyperparameter tuning

We use macro AP on the validation set for model selection in all cases. For k-NN-based ZSL, we choose *k* from {3,5,7}. For the bilinear ZSL, we perform a hyperparameter search among random combinations of learning rate (0.000001…0.1), optimizer (Adam, SGD, RMSprop), learning rate schedule (Exponential, Step, CosineAnnealing), momentum (0.95…0.9999), and the weight decay (0.00001…0.01). Finally, to measure the effect of initialization, unless otherwise stated, we train BZSM models three times and report the mean and standard deviation of the macro AP values.

### 3.2 DARKIN benchmark statistics

**Figure 2.**
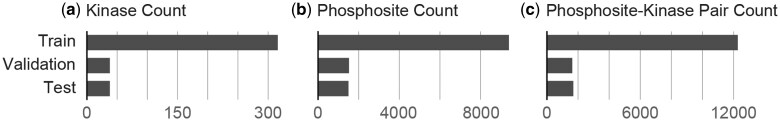
(a) The number of kinases. (b) The number of unique phosphosites. (c) The number of kinase–phosphosite pairs in each train, validation, and test folds of the default DARKIN split dataset.

**Figure 3.**
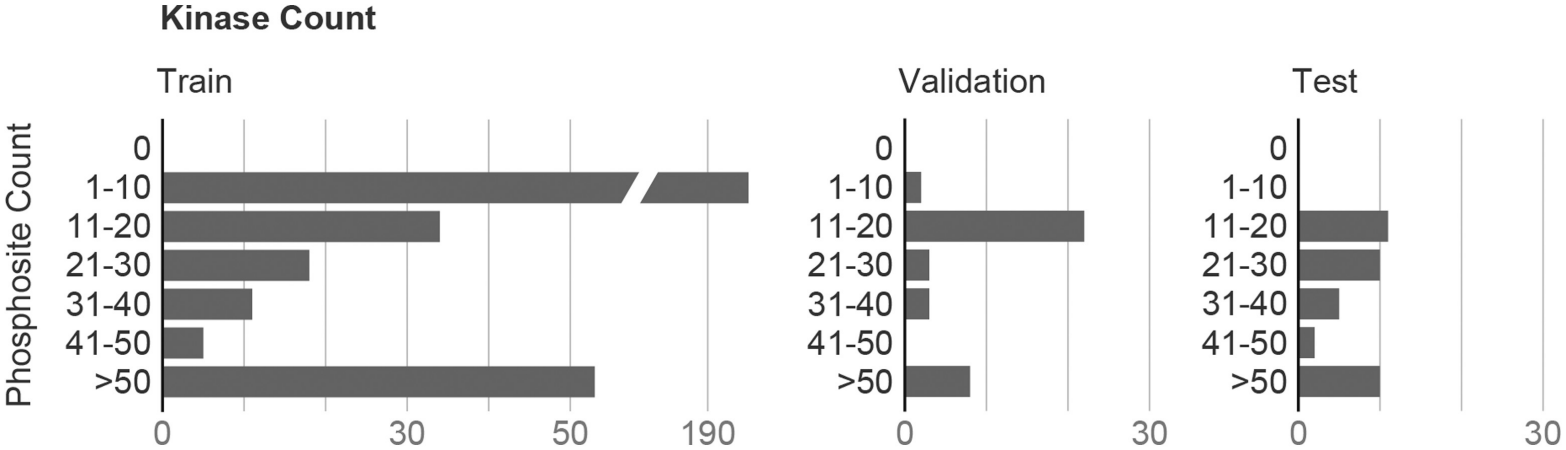
The histogram of the number of phosphosites associated with kinases in train, validation, and test sets in the default DARKIN split. See Section 2.4 for details.

**Figure 4.**
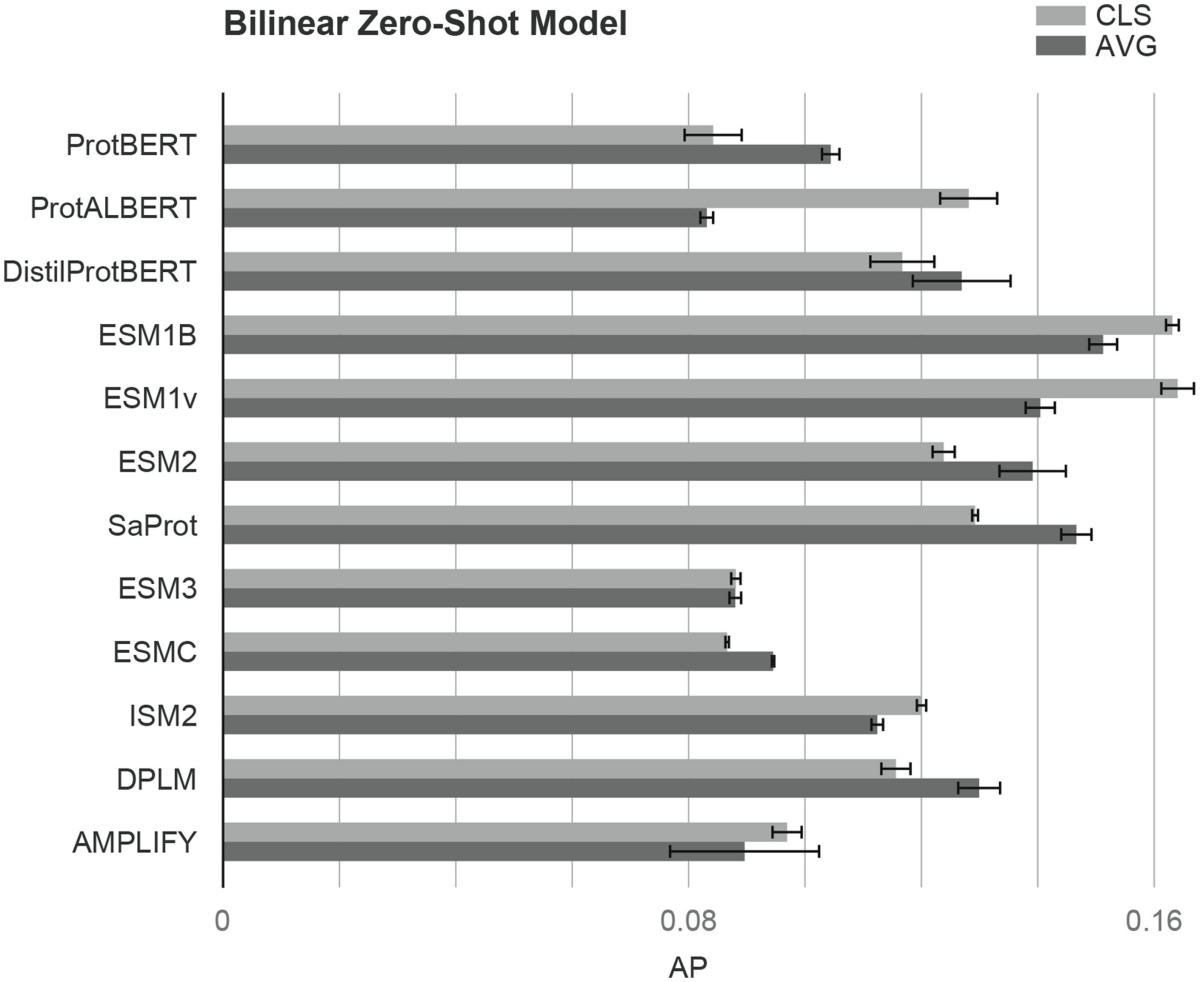
Performance comparison of BZSM models across different pLMs. The *x*-axis represents the average precision (AP) and the *y*-axis lists the evaluated pLMs. Light gray bars correspond to results obtained using the CLS token representation, while dark gray bars correspond to results obtained using the average of all token embeddings. Error bars indicate standard deviation across multiple runs.

We present four DARKIN splits (https://github.com/tastanlab/darkin and https://zenodo.org/records/16729884) for researchers. The experiments are conducted using DARKIN Split 1 unless otherwise specified. Therefore, we share the statistics for Split 1. The number of kinases, distinct phosphosites, and the phosphosite–kinase associations in the train, validation, and test sets are shown in Fig. 2. Furthermore, the histogram displaying the number of kinases associated with specific numbers of phosphosites is presented in Fig. 3. The balanced distribution of kinases according to kinase groups and the resulting kinase–phosphosite pair distribution can be analyzed in Fig. 2, available as supplementary data at *Bioinformatics* online, which results from the stratification strategy we used when splitting the kinase–phosphosite pairs. In addition to these statistics, further statistics such as the number of single-kinase and multi-kinase phosphosites (Fig. 3, available as supplementary data at *Bioinformatics* online), seen and novel sites in the test dataset (Fig. 4, available as supplementary data at *Bioinformatics* online), and the distribution of sites by the number of kinases they are associated with in the train, validation, and test sets (Fig. 5, available as supplementary data at *Bioinformatics* online) are accessible in the Supplementary text, available as supplementary data at *Bioinformatics* online.

### 3.3 Comparison of protein language models

We initially assess the effectiveness of pLM-based embeddings using both k-NN and BZSM methods. Table 2 presents macro AP scores obtained through the k-NN and BZSM when different pLM embeddings (detailed in Table 1) are used to represent the 15-mer around the phosphosite sequence and the kinase domain sequence. When employing pLM embeddings, we utilize embeddings sourced from the same pLM for both the phosphosite and kinase. To establish baseline performance, we also present results obtained with three sequence encoding methods: one-hot encoding, BLOSUM62, and NLF encoding (Section Evaluated Protein Language Models and Baseline Encodings). In both models, we observe that most pLM representations outperform the baseline encodings, indicating that they capture the protein sequences’ relevant characteristics better.

**Table 2.** Mean macro AP of 3-NN and BZSM using only pLM embeddings.

Embedding	AP (3-NN)	AP (BZSM)
OneHotEnc	0.0897	0.0634 ±0.0034
Blosum62	0.0897	0.0327 ±0.0008
NLF	0.0902	0.0419 ±0.0030
ProtVec	0.0808	0.0959 ±0.0010
ESM1B (cls)	0.1119	0.1631 ±0.0011
ESM1v (cls)	0.1121	**0.1640** ±0.0028
ESM2 (avg)	0.0957	0.1391 ±0.0057
Ankh-Large	0.1106	0.0840 ±0.0012
DistilProtBERT (avg)	0.0811	0.1269 ±0.0084
ProtBERT (avg)	0.0540	0.1044 ±0.0015
ProtAlbert (cls)	0.0915	0.1281 ±0.0049
ProteinBERT	0.1168	0.1236 ±0.0023
ProtGPT2	0.1054	0.1333 ±0.0020
ProtT5-XL	0.1172	0.1552 ±0.0011
SaProt (avg)	0.0973	0.1466 ±0.0026
TAPE	**0.1200**	0.1237 ±0.0018
ISM2 (cls)	0.0791	0.1200 ±0.0081
DPLM (avg)	0.1000	0.1299 ±0.0028
AMPLIFY (cls)	0.0873	0.0969 ±0.0025
ESM3 (cls)	0.0896	0.0881 ±0.0008
ESMC (avg)	0.0954	0.0945 ±0.0003
PTM-Mamba (phosphosite)^b^	0.0998	0.1218 ±0.0019

a For pLM with CLS versus average token embedding alternatives, the best performing one is shown.

b PTM-Mamba models use ESM2 embeddings for kinases. Since PTM-Mamba lacks a CLS token and includes special tokens for the phosphorylated residue, we used the embedding of that residue instead. The best results in each column are shown in bold. The versions of the models are specified in Table 4, available as supplementary data at *Bioinformatics* online.

The TAPE embeddings perform the best among the k-NN models (0.12 AP score). The ESM models and ProtT5-XL closely follow TAPE’s results (Table 2). In the BZSM models, however, the TAPE embeddings fall behind the ESM1B and ESM1v embeddings. The superior performance of TAPE in the k-NN could be due to it being a lower-dimensional vector (see Table 1). In BZSM, when employing the CLS token, ESM1B and ESM1v achieve over 0.16 macro AP. ProtT5-XL is the third close runner-up, and SaProt (cls) also performs well.

### 3.4 CLS token embedding versus averaging

Several pLMs provide a CLS token whose embedding is commonly used as the sequence summary (Devlin *et al.* 2019). However, it is not clear whether the CLS token or the average of all token embeddings provides a better summary for this task. The performance differences between these two alternatives are shown in Fig. 4, indicating that (i) the results can depend on this detail and (ii) the right option varies across the pLMs.

### 3.5 Incorporating additional kinase information

We augment the kinase sequence embedding vectors with additional information regarding kinase family hierarchy and EC classification. We encode these memberships as one-hot encoded vectors and append them to the sequence embedding vectors. Here, we experiment only with the BZSM since it outperforms the k-NN (The complete results obtained on the 3-NN with this additional kinase information are provided in Table 1, available as supplementary data at *Bioinformatics* online). Including each type of additional information individually enhances the performance of all models (Table 3), especially the inclusion of the kinase family information. The models based on ESM1B, ESM1v, and SaProt, using the CLS token embeddings, benefit the most and emerge as the best performers in this augmented case. These findings underscore that there is additional information in these kinase categorizations that cannot be captured solely with sequence information. The detailed list of results obtained with all pLMs obtained on the BZSM with this additional kinase information is provided in Table 2, available as supplementary data at *Bioinformatics* online.

**Table 3.** The BZSM performance trained with sequence embedding and other kinase information.^a^

Embedding	Base	+ Family	+ Group	+ EC	+ Family + Group + EC
OneHotEnc	0.0634	0.1107	0.0832	0.0802	0.1098
Blosum62	0.0327	0.0318	0.0310	0.0337	0.0323
NLF	0.0419	0.0391	0.0425	0.0400	0.0426
ProtVec	0.0959	0.1262	0.1129	0.1214	0.1354
ProtBERT (cls)	0.0842	0.1170	0.1077	0.1132	0.1273
ProteinBERT	0.1236	0.1506	0.1215	0.1367	0.1359
ProtT5-XL	0.1552	0.1701	0.1531	0.1674	0.1731
ESM1B (cls)	0.1631	**0.1740**	**0.1688**	**0.1680**	0.1769
ESM1v (cls)	**0.1640**	0.1737	0.1653	0.1652	0.1734
ESM2 (avg)	0.1391	0.1588	0.1453	0.1496	0.1638
DistilProtBERT (cls)	0.1167	0.1360	0.1292	0.1287	0.1441
ProtGPT2	0.1333	0.1476	0.1412	0.1419	0.1557
Ankh-Large	0.0840	0.1417	0.1135	0.1178	0.1594
ProtAlbert (cls)	0.1281	0.1269	0.1276	0.1285	0.1372
SaProt (cls)	0.1292	0.1696	0.1424	0.1434	**0.1800**
TAPE	0.1237	0.1379	0.1333	0.1310	0.1455
ISM2 (cls)	0.1200	0.1275	0.1260	0.1333	0.1374
DPLM (avg)	0.1299	0.1427	0.1318	0.1368	0.1420
AMPLIFY (avg)	0.0896	0.0968	0.0944	0.0969	0.1066
ESM3 (cls)	0.0881	0.1484	0.1220	0.1238	0.1611
ESMC (cls)	0.0866	0.1672	0.1136	0.1401	0.1754
PTM-Mamba (phosphosite)^b^	0.1218	0.1432	0.1292	0.1346	0.1471

a The mean macro APs are shown. Of CLS and embedding averaging, only the best-performing model results are listed.

b PTM-Mamba models utilize ESM2 embeddings for kinases. Since PTM-Mamba lacks a CLS token and includes special tokens for the phosphorylated residue, we used the embedding of that residue instead. The best results in each column are  shown in bold.

### 3.6 Comparing the best-performing pLMs on different random DARKIN splits

As ESM1B and SaProt emerge as the two top-performing pLMs when paired with the BZSM model (Table 3), we further evaluated their performance on three additional random splits of the DARKIN dataset to facilitate a more comprehensive comparison between these two pLMs. While both models demonstrate competitiveness, SaProt consistently outperforms ESM1B slightly across all runs on these four different splits (Table 4). The performance of SaProt underscores the added value of structural information.

**Table 4.** The mean macro AP scores at multiple levels (family, group, phosphosite) for the two best-pLMs, ESM1B (Family + Group + EC) and SaProt (Family + Group + EC), on four random DARKIN splits for the BZSM.^a^

Split	Embedding	AP	Phospho-site AP	Family AP	Group AP	Masked Group AP
Split 1	ESM1B (cls)	0.1769	0.2830	0.2278	**0.3959**	**0.4054**
	SaProt (cls)	**0.1800**	**0.3053**	**0.2384**	0.3903	0.3868
Split 2	ESM1B (cls)	0.1536	0.2747	0.1989	**0.3689**	0.3644
	SaProt (cls)	**0.1599**	**0.2929**	**0.2087**	0.3649	**0.3702**
Split 3	ESM1B (cls)	0.1531	0.2951	0.1987	**0.3747**	0.3508
	SaProt (cls)	**0.1627**	**0.3142**	**0.2104**	0.3663	**0.3598**
Split 4	ESM1B (cls)	0.1652	0.3118	0.2142	0.3969	0.3563
	SaProt (cls)	**0.1690**	**0.3482**	**0.2205**	**0.4069**	**0.3674**

a The best performing results for each split comparison are shown in bold.

### 3.7 Extended evaluation of kinase family, group, and phosphosite predictions

We evaluated model performance on all DARKIN splits using macro AP at multiple levels: family AP, where kinase predictions are aggregated by their families; group AP, where kinase predictions are aggregated by their groups; and phosphosite AP, which evaluates precision for phosphosite-specific predictions. We also calculated the hit@k accuracy for these models (Table 3, available as supplementary data at *Bioinformatics* online). In all these metrics, SAProt shows slightly better performance.

Additionally, we introduced a metric, Masked Group AP, which focuses on precision within the true group by excluding irrelevant kinases from the predictions. This metric works by masking logits for kinases outside the ground-truth kinase group, effectively setting them to negative infinity. This metric simulates a scenario where the model perfectly identifies kinase groups and predicts within the group. This allows us to measure the model’s ability to rank kinases accurately within groups. Our findings, summarized in Table 4, show that Masked Group AP significantly outperforms standard AP, with values greater than twice those of standard AP calculated over all classes. This improvement demonstrates the strong impact of incorporating group-level information, suggesting that if kinase groups could be predicted accurately—whether by this or a separate model—the performance jump in kinase ranking could be substantial. This insight suggests a promising direction for future research, where accurate group predictions could serve as a basis for refining kinase-level predictions.

### 3.8 Fine-tuning of phosphosite and kinase encoders

To evaluate if task-specific fine-tuning improves the performance, we extended the original BZSM setup—which keeps phosphosite and kinase embeddings fixed and only learns the compatibility matrix—by adding four fine-tuning variants and evaluating them using the two well-performing pLMs, ESM1B and ProtT5-XL. First, we allowed end-to-end fine-tuning of the phosphosite encoder while keeping the kinase encoder frozen and still learning the compatibility matrix W. Next, we gradually unfroze the kinase encoder, reinitializing and training either its final transformer block or, in a deeper variant, the last two blocks, so that both phosphosite and kinase representations could adapt jointly with the compatibility matrix *W*. Finally, in the fourth variant we experimented with a fully shared encoder that produces both phosphosite and kinase embeddings; here, the entire model is fine-tuned jointly, and compatibility is computed via a simple dot product, eliminating the need for W. Each regime was tested with two kinase representations: sequence-only embeddings and appending the sequence embeddings with family, group, and EC information vectors.

As presented in Table 5, fine-tuning the pLM encoders does not guarantee improved performance. Instead, the results were inconsistent across different configurations. For the ESM1b model, the highest performance was AP of 0.1911, achieved by reinitializing the final two layers of both the phosphosite and kinase encoders using the full set of kinase features. However, this represents only a marginal improvement over other configurations and comes at a notable computational cost. Similarly, the ProtT5-XL model saw a slight performance increase to an AP of 0.1800 when reinitializing the last layer of both encoders. Notably, most other fine-tuning strategies resulted in a decrease in performance for both models.

**Table 5.** Experiments on fine-tuning ESM1b and ProtT5-XL, in which we employ transformers to fine-tune either the phosphosite model or the phosphosite and kinase model simultaneously.^a^

Transformer Configuration	BZSM	Dot product	Kinase features	AP	
				**ESM1B**	**ProtT5-XL**
Fully fine-tune transformer, remove BZSM		✓	Seq	0.0996	0.1593
Fully fine-tune phosphosite model, freeze kinase model	✓		Seq	0.1622	0.1298
Reinitialize last layer of phosphosite and kinase models	✓		Seq	0.1852	0.1375
Reinitialize last two layers of phosphosite and kinase models	✓		Seq	0.1283	0.1285
Fully fine-tune phosphosite model, freeze kinase model	✓		Seq, Family, Group, EC	0.1638	0.1765
Reinitialize last layer of phosphosite and kinase models	✓		Seq, Family, Group, EC	0.1669	**0.1800**
Reinitialize last two layers of phosphosite and kinase models	✓		Seq, Family, Group, EC	**0.1911**	0.1575

a As a baseline evaluation, we remove BZSM and evaluate zero-shot predictions by the dot product of learned kinase and phosphosite representation. ESM1b embeddings are obtained using CLS token representation, while ProtT5-XL embeddings are obtained using the average of all token embeddings. Training and evaluation protocols are the same for both pLMs. The best performing models' results are shown in bold.

We explored other fine-tuning strategies. To arrive at phosphosite-aware and kinase-aware pLMs, we conducted comprehensive experiments in which we fine-tuned pLMs with kinase- and phosphorylation-related auxiliary tasks. These tasks include (i) phosphorylation prediction, given a potential phosphosite and its surrounding sequence, the model is trained to predict if this site is phosphorylated or not. (ii) Kinase group prediction, given the kinase domain sequence, predicting the group of the given kinase. This is a multi-class classification task. (iii) Contrastive learning on family/group relations. In this task, the model should learn the kinase family/group relationships in a contrastive learning setup. We present the dataset, experimental methods, and the results in the Section 4, available as supplementary data at *Bioinformatics* online. None of these phosphosite and kinase fine-tuning strategies match the performance of the end-to-end fine-tuning presented above (AP score of 0.1911) obtained by reinitializing the last two layers of the ESM1B model.

## 4 Conclusion

Focused on the zero-shot task of assigning phosphosites to understudied dark kinases, DARKIN offers a novel benchmark for evaluating pLMs. As it is easy to fall into the data leakage pitfalls in these types of problems, as raised and discussed in drug-target prediction (Chatterjee *et al.* 2023), drug synergy prediction (Beyza Çandır *et al.* 2025), in genomics (Whalen *et al.* 2022), or link prediction (Brière *et al.* 2025), it is important to evaluate the models in robust evaluation frameworks to assess the generalization of these models (Bernett *et al.* 2024). In this work, the train, validation, and test splits are carefully designed to follow ZSL and kinase-related issues. We evaluate the pLMs’ representation capabilities in this problem using two zero-shot classifiers. Our results demonstrate the superior performance of the ESM models, the ProtT5-XL, and the SaProt models.

Based on our results using the DARKIN dataset, dark kinase–phosphosite prediction remains a highly challenging task for the current pLMs. The highest AP score achieved was 0.1911 using fine-tuning pLMs, which considerably outperforms random guessing (0.03 by averaging AP over 1000 runs of randomly generated ranking of kinases for a given site), but can be considered low overall. The low performance could be due to several reasons. There are challenging cases where the phosphosite sequences are almost identical, but the associated kinase sets for these phosphosites differ. This difference could be due to a true biological difference that can be explained by a structural or functional difference (a required interaction partner or the same cellular localization), or it could also be an issue of data incompleteness. While some kinase–phosphosite pairs are truly associated, they might not have been experimentally studied and therefore are not reported as associated pairs. We should also note that the performances in a deployed model of dark–kinase associations are likely to be higher. To ensure a sufficient number of examples in the evaluation, as explained in Section 2, we switched the light and dark kinases in the train and test sets. In this way, the test set included the well-studied kinases with more examples, and the training set included the understudied kinases. While this strategy is useful for benchmarking purposes, it poses a challenge in training, as the training data contains many kinases with few examples. Since the deployed model uses the well-studied kinases as well, it is likely to have better predictive performance.

In this study, we excluded fusion kinases and non-canonical kinase isoforms in constructing the datasets. This was due to the lack of annotation of their kinase domains in some cases and the low number of known associated phosphosites, which made it difficult to reliably evaluate the models’ performance on these kinases. These kinase forms can play crucial roles in disease contexts such as cancer, where gene fusions or isoform-specific events give rise to novel or dysregulated signaling activities (Stransky *et al.* 2014, Gonzalez and McGraw, 2009). Thus, zero-shot predictions coupled with experimental validation on these kinases can open new avenues for understanding the functional impact of isoforms and oncogenic fusions.

The study focused on the ZSL framework. Another promising direction and interesting benchmark is the few-shot learning problem, in which the model leverages the few known phosphosites of the kinases during the training. The current DARKIN dataset can be modified for this setup easily. We hope this novel benchmark will facilitate comprehensive evaluations of pLMs and dark kinase prediction models, contributing to protein biology research.

## Supplementary Material

btaf480_Supplementary_Data

## Data Availability

We present four DARKIN splits (https://github.com/tastanlab/darkin and https://zenodo.org/records/16729884) for researchers.
